# Posterior Mediastinal Adenomatoid Tumor: A Case Report and Review of the Literature

**DOI:** 10.1155/2016/6898526

**Published:** 2016-05-08

**Authors:** Vishwas Parekh, Thomas Winokur, Robert J. Cerfolio, Todd M. Stevens

**Affiliations:** ^1^Department of Pathology, University of Alabama at Birmingham, Birmingham, AL 35249, USA; ^2^Department of Surgery, University of Alabama at Birmingham, Birmingham, AL 35249, USA

## Abstract

Adenomatoid tumor is an uncommon benign neoplasm of mesothelial differentiation that distinctively arises in and around the genital organs. In rare instances, it has been described in extragenital locations. There have been only two reports documenting its occurrence in the anterior mediastinum, and no reports documenting its occurrence in the posterior mediastinum. We report the first case of posterior mediastinal adenomatoid tumor. A 37-year-old Caucasian woman presented with symptoms of bronchitis. Imaging studies identified a 2.0 cm posterior mediastinal mass abutting the T9 vertebral body, clinically and radiologically most consistent with schwannoma. Histologic sections revealed a lesion composed of epithelioid cells arranged in cords and luminal profiles embedded in a fibrotic to loose stroma and surrounded by a fibrous pseudocapsule. Lesional cells showed vacuolated eosinophilic cytoplasm and peripherally displaced nuclei with prominent nucleoli. There was focal cytologic atypia but no mitotic figures or necrosis was identified. The lesional cells expressed cytokeratin, calretinin, and nuclear WT1 but were negative for PAX8, TTF1, p53, chromogranin, CD31, and CD34, and Ki67 showed <2% proliferation rate, diagnostic of adenomatoid tumor. Three years after resection, the patient is in good health without tumor recurrence. Thus, our encounter effectively expands the differential diagnosis of posterior mediastinal neoplastic entities.

## 1. Introduction

The mediastinum is delimited proximally by the thoracic inlet, distally by the diaphragm, and laterally by the pleural cavities. It is arbitrarily divided into anterior, middle, and posterior compartments. The anterior compartment contains the thymus, lymph nodes, and occasionally substernal extensions of thyroid and parathyroid glands; the middle compartment contains the heart, great vessels, lymph nodes, trachea, and phrenic and vagus nerves; and the posterior compartment contains the esophagus, descending aorta, thoracic duct, lymph nodes, sympathetic trunks, and spinal ganglia. Additionally, the anterior and posterior compartments contain the intercostal vessels and nerves. These anatomic differences are reflected in the limited variety of neoplasms that arise in each of these three compartments, with the majority of tumors in the posterior compartment being of neurogenic differentiation (Box 1).

Adenomatoid tumor is a relatively uncommon benign neoplasm of mesothelial lineage that primarily arises in the uterus and testes and surrounding adnexal soft tissues during the reproductive age [1, 2]. Extragenital adenomatoid tumors are rarer and have been documented in a variety of locations such as adrenal gland, liver, small intestine, omentum, heart, lymph node, and pleura [3–11]. Of the four reported cases of adenomatoid tumors of pleura, three occurred in association with visceral pleural surfaces of various lung lobes [9, 10] and one occurred in connection with the parietal pleura of right lung [11]. Importantly, none of these masses were located in the posterior mediastinum. Two case reports documented the occurrence of adenomatoid tumor in the anterior mediastinum, both in connection with the pericardial reflections [12, 13]. Here, we describe the first case of adenomatoid tumor located in the posterior mediastinum, thereby expanding the limited list of entities that arise in this location.

## 2. Materials and Methods

The specimen was grossly examined and sectioned. A representative section was processed and stained with hematoxylin and eosin (H&E) for frozen section interpretation. The remainder of the mass lesion and the frozen section remnant were fixed in 10% neutral buffered formalin, routinely processed, and embedded in paraffin for histologic processing. Tissue sections were stained with H&E for routine histologic examination. The immunostaining was accomplished with a Ventana (Ventana Autostainer Benchmark Ultra, Ventana Inc., Tucson, AZ) or Dako semiautomated immunostainer (Dako Autostainer Link 48, Carpinteria, CA). Immunohistochemical examination of blocks was preformed according to the manufacturer's manual. Briefly, 4 *μ*m sections were obtained from formalin-fixed, paraffin-embedded block preparations. Heat induced epitope retrieval with 0.02 M concentration of citrate buffer (pH 9.0 or pH 6.0) in a heater at 97°C for 20 minutes was applied. The following antibodies were tested: pancytokeratin (AE1/AE3/PCK26, Ventana Medical Systems, Inc., Tucson, AZ, USA), TTF1 (Clone 8G7G3, Dako), PAX8 (Cat# CDP379 CK, rabbit polyclonal, 1 : 50 dilution, Biocare Medical, Concord, CA, USA), p53 (Clone Bp53-11, Ventana), MIB/Ki67 (Clone 30-9, Ventana), calretinin (Clone SP65, Ventana), WT1 (Clone 6F-H2, Dako), chromogranin (Clone LK2H10, Ventana), CD31 (Clone 1A10, Dako), and CD34 (Clone Ab-1, Dako). Except for PAX8, all other antibodies were purchased prediluted by the manufacturer. The chromogen diaminobenzidine tetrachloride was used to visualize the antibody-antigen complex. The tissue was counterstained with hematoxylin. Appropriate positive and negative control slides were reviewed.

## 3. Clinical History

**Figure 1:**
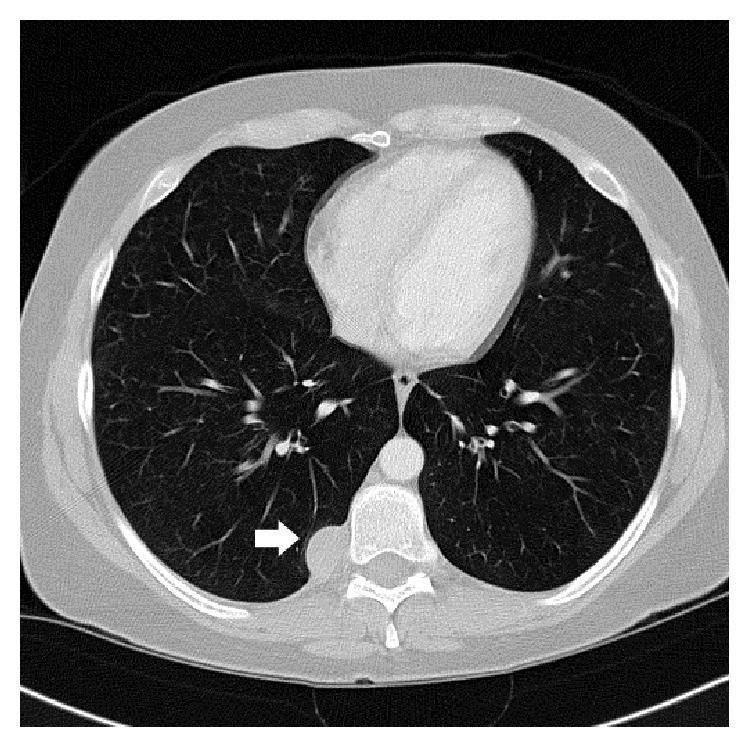
CT scan with contrast demonstrates a 2.0 cm posterior mediastinal mass abutting the right T9 rib and vertebral body (arrow).

A 37-year-old Caucasian woman with a 20-year history of cigarette smoking, a family history of lung cancer, and no other significant past medical history presented with symptoms of bronchitis. A chest X-ray and a CT scan identified an incidental 2.0 cm posterior mediastinal mass abutting the T9 vertebral body (Figure 1), clinically and radiologically most consistent with schwannoma. A minimally invasive surgical approach showed no adjacent pleural thickening or multifocal nodules. The mass lesion was completely excised and sent for an intraoperative frozen section interpretation. Three years after resection, the patient is in good health without tumor recurrence.

## 4. Pathologic Findings

The gross examination revealed an ovoid, tan-white, completely encapsulated, rubbery mass that measured 2.3 × 2.2 × 1.7 cm. Frozen section examination showed cytologically bland epithelioid cells arranged in cords and luminal configuration, ruling out the possibility of schwannoma. A definitive diagnosis was not rendered during frozen section evaluation.

**Figure 2:**
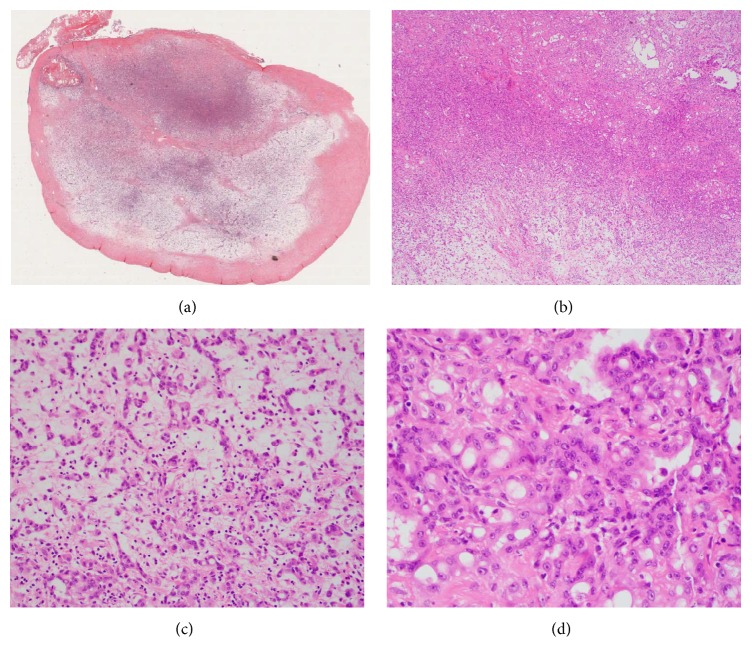
(a) The neoplasm was confined to a fibrous pseudocapsule. (b) Hypercellular and hypocellular areas were present. (c) and (d) The neoplasm grew as cords and luminal profiles and contained mildly pleomorphic nuclei with prominent nucleoli.

**Figure 3:**
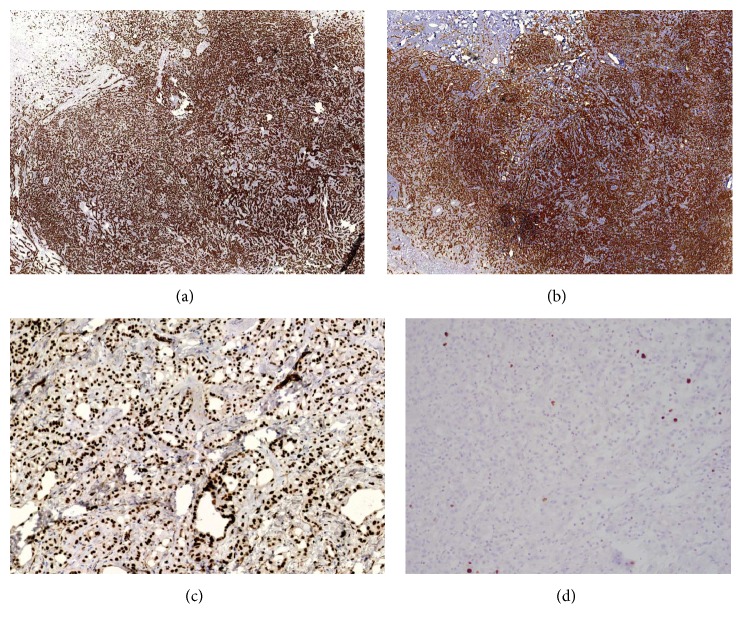
The mesothelial nature of the neoplasm was confirmed by its expression of pancytokeratin (a), calretinin (b), and nuclear WT1 (c). Ki-67 (d) showed a <2% proliferation rate. A diagnosis of adenomatoid tumor was made.

On permanent sections, the lesion was confined to a fibrous pseudocapsule (Figure 2(a)) and was composed of epithelioid cells arranged in cords and luminal profiles. Some areas of the neoplasm showed tightly packed luminal profiles creating a hypercellular appearance, while other areas showed neoplastic cells arranged as cords and single cells in a loose stroma creating focal hypocellular areas (Figures 2(b)–2(d)). Cytologically, the neoplastic cells contained mildly pleomorphic nuclei with vesicular chromatin, prominent nucleoli, and vacuolated eosinophilic cytoplasm (Figures 2(c)-2(d)). No mitotic figures were observed and the Ki67 proliferative rate was <2%. No infiltrative features were seen. By immunohistochemistry, the lesional cells expressed pancytokeratin, calretinin, and nuclear WT1 in a strong and diffuse manner (Figures 3(a)–3(c)) but were negative for TTF1, CD31, CD34, PAX8, p53, and chromogranin, confirming mesothelial differentiation. Among the mesothelial lesions, the differential diagnosis would include reactive mesothelial hyperplasia, adenomatoid tumor, and malignant mesothelioma, as malignant mesothelioma may also show adenomatoid-like areas [14]. The gross nodular configuration of the lesion and an adenomatoid histologic pattern, devoid of significant inflammatory background, supported a neoplastic rather than a reactive process. A lack of diffuse pleural involvement, presence of a gross circumscription and encapsulation, lack of necrosis and infiltrative features such as capsular or lymphovascular invasion, an absence of marked cytologic atypia, and a proliferation rate of <2% as assessed by a MIB-1 (Ki67) immunostain (Figure 3(d)) all favored a diagnosis of adenomatoid tumor. Although mesothelioma may also have bland cytologic features and a low mitotic index, the surgical findings and the long clinical follow-up without recurrence in combination with the other features of the neoplasm essentially exclude a diagnosis of mesothelioma. A final diagnosis of adenomatoid tumor was rendered.

## 5. Discussion

A wide variety of pathologic entities, both neoplastic and nonneoplastic, arise in the mediastinum. This necessitates a systematic approach to diagnosis, including a careful consideration of the patient's age, gender, clinical history, results of the imaging studies, clinical laboratory tests, and finally, the histopathologic examination of a biopsy or resection specimen, complimented by ancillary studies such as immunohistochemistry, flow cytometry, and molecular testing, as appropriate. This process is greatly aided by the knowledge of the limited number of pathologic entities that are known to occur in each of the mediastinal compartments, allowing the practitioners to follow an algorithmic decision process and quickly narrow down the diagnoses to the correct one.

As depicted in Box 1, the list of neoplasms that are currently known to occur in the posterior mediastinum does not include an adenomatoid tumor, until now. That being the case, when clinicians, radiologists, and pathologists evaluate a mass-like lesion in the posterior mediastinum, they are very likely to not entertain the possibility of this entity in their diagnostic consideration, with a consequent delay in reaching a diagnosis or a real possibility of misdiagnosis.

Adenomatoid tumors are solitary, circumscribed neoplasms comprised of cytologically bland cuboidal epithelioid cells arranged in cords and gland-like/tubular structures in a fibrous or fibromyxoid stroma, often rendering a microcystic appearance. Individual tumor cells show peripherally displaced nuclei with vesicular chromatin, inconspicuous to prominent nucleoli, and abundant eosinophilic or vacuolated cytoplasm. Signet ring–like cells and pseudolipoblasts may be present. Nuclear pleomorphism is minimal to absent; mitotic figures are absent or very rare; atypical mitoses and tumor necrosis are absent; and there are no infiltrative features. Immunohistochemically, the tumor cells express cytokeratin, calretinin, D2-40, thrombomodulin, and nuclear WT1 but are negative for Ber-EP4, CEA, CD15, and CD34. Ki-67 (MIB-1) shows a labeling index of only 1% to 2%. This is helpful to distinguish it from malignant mesothelioma with microcystic appearance, which shows a high Ki-67 labeling index (>50%) [14–17]. Ultrastructurally, true to their mesothelial character, tumor cells show branching plasmalemmal microvilli and prominent intercellular attachment complexes [8].

In summary, we document the first case of adenomatoid tumor arising in the posterior mediastinum, effectively expanding the number of pathologic entities that are known to occur in this location. Although rare, this case serves as an instruction to the clinicians, radiologists, and pathologists to include adenomatoid tumor in the differential diagnosis when working up a posterior mediastinal tumor.

## Figures and Tables

**Box 1:**
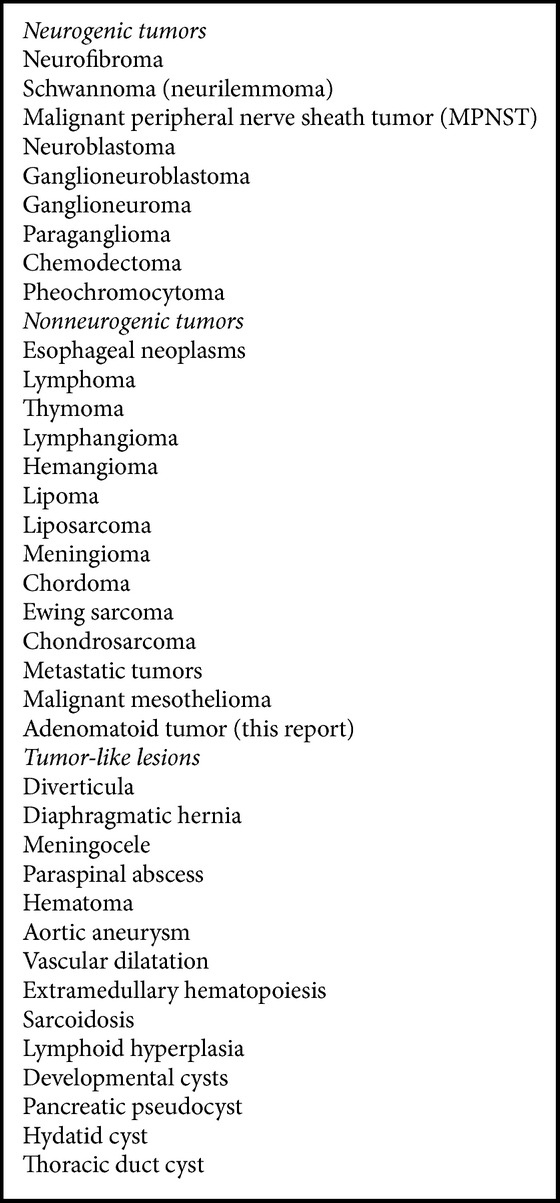
Tumor and tumor-like lesions of the posterior mediastinum.
